# Age-related macular degeneration associated with optic disc drusen

**DOI:** 10.3389/fopht.2025.1620616

**Published:** 2025-07-03

**Authors:** Anas Alkhabaz, Min Young Kim, Rishita Pujari, Jamie Zhang, Yulan Ren, Loh-Shan Bryan Leung, Yaping Joyce Liao

**Affiliations:** ^1^ Department of Ophthalmology, Stanford University School of Medicine, Stanford, CA, United States; ^2^ Oakland University William Beaumont School of Medicine, Rochester, MI, United States

**Keywords:** optic disc drusen, age-related macular degeneration, multimodal imaging, macular drusen, calcification

## Abstract

**Objective:**

The aim of this study was to investigate the risk of age-related macular degeneration (AMD) in association with optic disc drusen (ODD).

**Design:**

This was an observational, cross-sectional study.

**Participants:**

Participants were consecutive patients with and without ODD from the neuro-ophthalmology clinic. Ten patients with concomitant ODD-AMD were sub-analyzed.

**Methods:**

The two cohorts were identified from a prospectively recruited dataset between July 2022 and June 2024. Patients received formal diagnoses of ODD and AMD after ophthalmic and imaging assessment. A logistic regression model was utilized in calculating AMD risk to account for demographic differences.

**Results:**

A total of 94 patients with ODD (median age: 44 [Q1: 20, Q3: 69], 64% women) and 100 patients without ODD (median age: 60 [Q1: 44, Q3: 69], 48% women) were identified. AMD was observed in 9.6% and 3% of the ODD and non-ODD cohorts, respectively. The risk of AMD was higher in the ODD group (OR = 3.93, 95% CI: 0.89–21.85, *p* = 0.084). Although the association was not statistically significant, a logistic regression model attributed that to the age difference between the two cohorts. Of the 10 patients with ODD-AMD, 70% had a family history of AMD. These patients were all Caucasians and had a median age of 75 years (range: 56–91); 70% were women. Only 30% were smokers. On optic disc imaging, 70% of eyes demonstrated moderate-to-severe ODD.

**Conclusion:**

Patients with ODD might be at a higher risk of AMD compared to patients without ODD, and AMD screening might be warranted. A family history of AMD is often present, indicating shared genetic risk factors.

## Introduction

Macular and optic disc drusen (ODD) are the most common extracellular deposits in the eye. Macular drusen are lipid-rich deposits that accumulate under the retinal pigment epithelium (RPE), most commonly in age-related macular degeneration (AMD), which accounts for visual impairment in 8.69% of the global population aged 45 to 85 years (1, 2). Recently, AMD has been reported to be associated with ectopic calcification, which is an independent risk factor for AMD progression (3). In fact, Thompson et al. et al. suggested that an element of calcium might be present in all subretinal deposits (4).

ODD, the most common cause of pathologic mineralization of the optic nerve, are calcified concretions in the unmyelinated anterior optic nerve. It occurs in 2% of the general population, most commonly in Caucasians and sometimes in an autosomal dominant fashion in families (2, 5). ODD is most commonly bilateral (66%) and occurs more frequently in women (62%). In symptomatic patients, ODD most commonly presents with visual field defects, especially when the drusen are visible ophthalmoscopically. In the absence of associated macular degeneration or ischemic optic neuropathy, central vision is usually preserved (6, 7).

Lorentzen briefly reported the first fundus image of a patient with both ODD and AMD (ODD-AMD) in 1961 (8). In 2004, Scartozzi and Tasman reported another case of ODD-AMD with familial ODD (9). No other reports have been published since then. Hence, to understand this co-occurrence, we investigate the risk of AMD among an ODD cohort from the neuro-ophthalmology clinic. We further analyze the optic nerve and macular drusen features through multimodal imaging.

## Methods

### Participant information

To determine the frequency of AMD among patients with ODD and to compare the clinical and imaging features, we performed a retrospective analysis from a dataset of 650 prospectively recruited patients with various optic neuropathies, including ODD, at Byers Eye Institute, Stanford University, USA. The Stanford Institutional Review Board approval was obtained and electronic medical records were reviewed for each patient in accordance with the Declaration of Helsinki and the Health Insurance Portability and Accountability Act.

We identified 94 consecutive patients with ODD and a cohort of 100 consecutive neuro-ophthalmology patients without ODD presenting for evaluation of optic neuropathy (July 2022 and June 2024). The non-ODD group included patients presenting for evaluation of optic neuropathy. Among these, the most frequent diagnosis was ischemic optic neuropathy in 38%. Other diagnoses included papilledema, optic atrophy, Leber’s hereditary optic neuropathy, mitochondrial deletion syndrome, nonspecific visual disturbances, and others. An additional patient with ODD-AMD met our inclusion criteria through chart review, leading to a total of 10 patients (20 eyes) with ODD-AMD. All patients had comprehensive ophthalmic examination and multimodal imaging. AMD was diagnosed and classified into early, intermediate, or late (dry form with geographic atrophy or exudative form with neovascularization) stages based on the Beckmann Classification for AMD (10). The diagnosis for ODD was established based on the EDI-OCT images, the gold standard for diagnosis according to The Optic Disc Drusen Studies Consortium. Furthermore, the drusen were categorized into superficial and buried in relation to Bruch’s membrane (11). Patients with nonspecific retinal deposits, retinal dystrophies, or neuro-ophthalmic diseases that can impact analysis were excluded.

### Multimodal imaging

We performed multimodal imaging using FAF and OCT. FAF was acquired using an ultra-wide field Optos system (Optos PLC, Dunfermline, Scotland). Spectral-domain OCT images were acquired for the optic disc and macula using Cirrus HD-OCT (Carl Zeiss Meditec Inc., Jena, Germany). We acquired an Optic Disc Cube (200 × 200) with 200 horizontal scan lines and a Macular Cube (512 × 128) with 128 horizontal scan lines. We also acquired the 97-line spectral-domain EDI-OCT scans of the optic nerve (Spectralis HRA; Heidelberg Engineering, Heidelberg, Germany) (11).

### Quantification of drusen reflectivity on OCT

**Figure 1:**
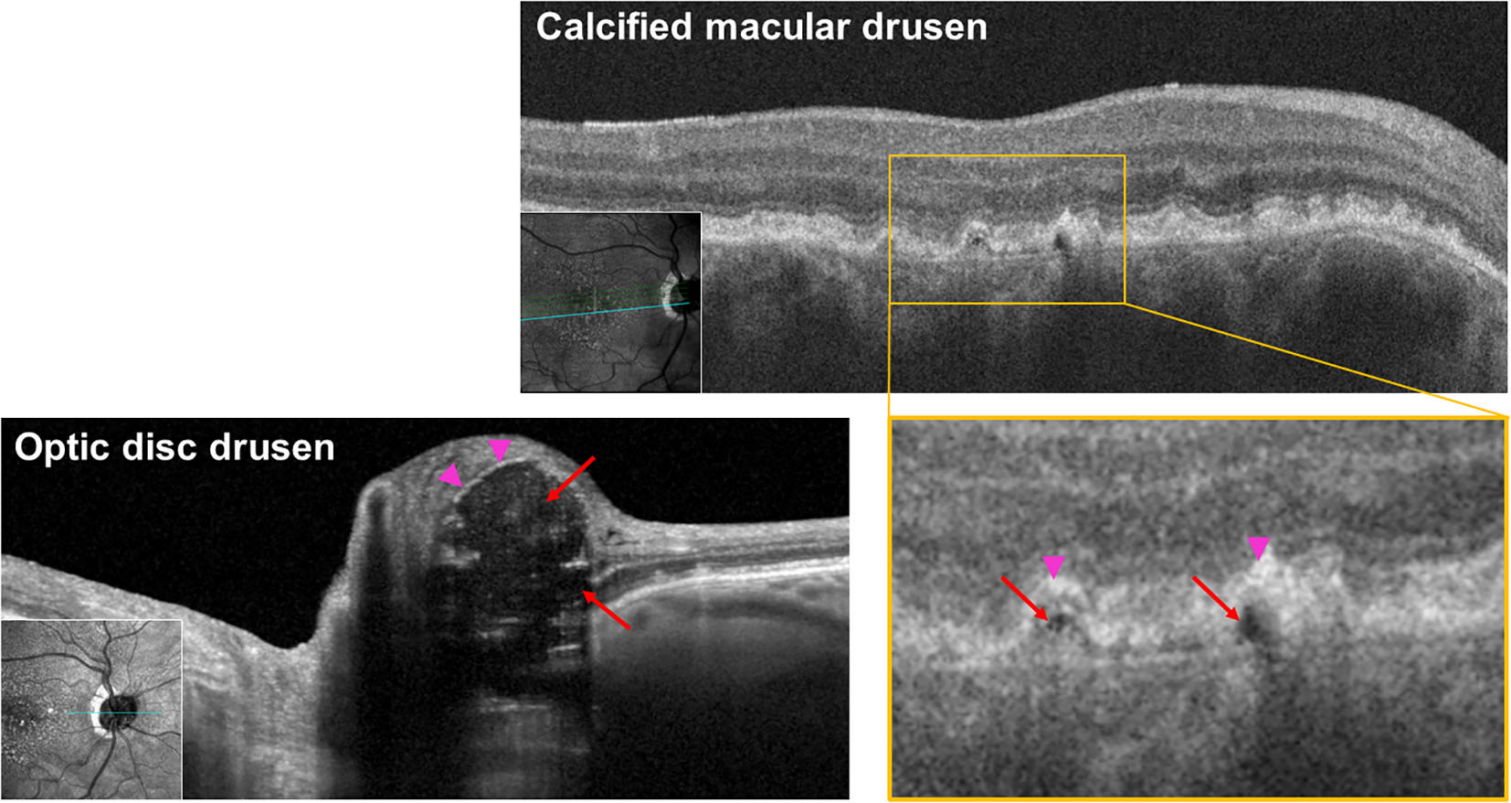
Infrared OCT B-scan of calcified macular drusen and optic disc drusen at 815 nm showing hypodense core in both locations, consistent with ectopic calcification. Red arrows: hyporeflective core; pink arrowheads: hyperreflective cap.


We identified 26 likely calcified macular drusen, 34 noncalcified macular drusen, and 19 ODD from the study eyes based on published criteria (3). Then, we measured the near-infrared light reflectivity of the drusen core, the drusen cap, and the difference between them using the image processing software, FIJI (An enhanced version of ImageJ). We used a previously described method with some modifications tailored to our parameters (12–15). **Figure 1** illustrates the regions defined as drusen core and cap in ODD and macular drusen. Reflectivity was defined as the average gray value of the selected pixels, and a value of 0 corresponds to pure black and a value of 255 corresponds to pure white. To eliminate the signal variation between the OCT images, average gray values from quadrangular areas of the vitreous and retinal nerve fiber layer (RNFL) were used for standardization. We calculated the average reflectivity of the drusen core (a region of low to no reflectivity). The drusen cap (a region of high reflectivity) signal was calculated as the average of the top five pixels above the core. We calculated the difference between the drusen cap and drusen core for each macular drusen using the formula:

$$\text{Cap}-\text{Core score }=\frac{\left(\text{Cap}-\text{Core }\right)-\text{vitreous reflectivity}}{\text{RNFL reflectivity}-\text{ vitreous reflectivity}}$$

To calculate macular drusen disease burden, defined as the total volume of macular drusen per eye, we manually outlined all macular drusen on the SD-OCT Macular Cube 512 × 128 scans (128 OCT B-scan sections/eye) and calculated drusen volume using ImageJ. To minimize variability, this process was done by a single investigator.

### Statistical analysis

Statistical analysis was performed using R 2023.12.1 + 402 (https://www.r-project.org/). Quantitative variables were reported as mean and standard deviation and qualitative variables were reported as a percentage. The odds ratio was used to calculate the risk of AMD among ODD and non-ODD cohorts. A logistic regression model was used to account for possible confounding variables between the two cohorts. The Cap-Core scores in each group were tested for normality of distribution using the Shapiro–Wilk test, and the mean and standard deviation were reported. GraphPad Prism (Prism 10.2.2; GraphPad Software, Inc., La Jolla, CA) was then used to compare the means of the three groups using one-way ANOVA and *post hoc* Tukey test. *p*-values of less than 0.05 were considered statistically significant.

## Results

**Figure 2:**
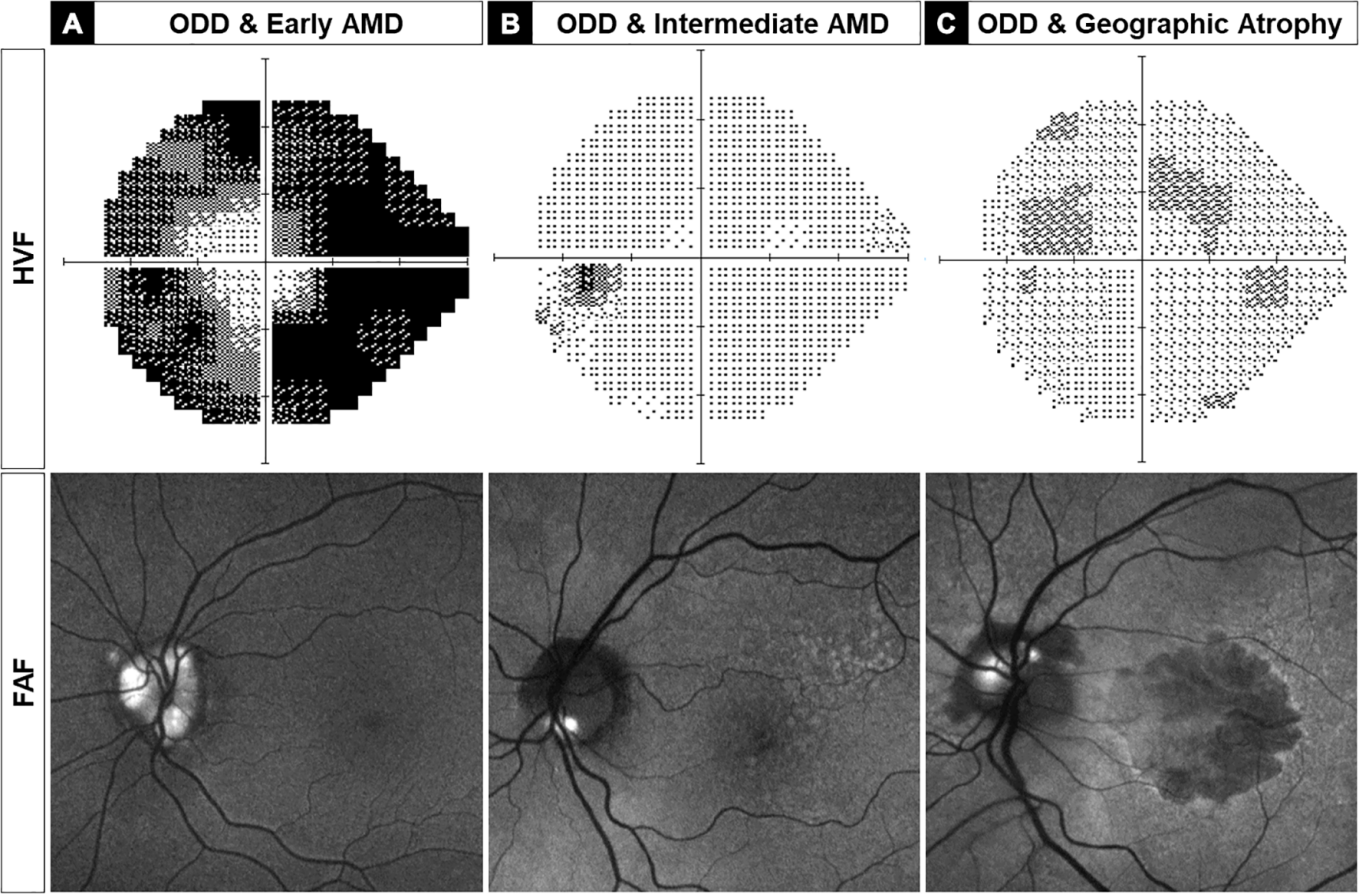
Three left eyes are representative examples of optic disc drusen-associated age-related macular degeneration (ODD-AMD) showing the range of vision loss on static perimetry (top) and severity of ODD and AMD on fundus autofluorescence imaging (FAF, bottom). **(A)** A 72-year-old man with severe ODD and severe visual field constriction, consistent with severe optic neuropathy. **(B)** A 77-year-old woman with good visual function with morphometric features of intermediate AMD and superficial ODD in the inferior optic disc border. **(C)** A 72-year-old woman with severe decrease in visual acuity and mild paracentral visual field loss, associated with severe AMD with geographic atrophy and superficial ODD in superior and nasal quadrants. AMD, age-related macular degeneration; HVF, Humphrey visual field; FAF, fundus autofluorescence.

Three representative examples of ODD-AMD eyes are shown in **Figure 1**: a 72-year-old man with severe ODD and mild AMD (**Figure 2A**), a 77-year-old woman with intermediate-stage AMD and relatively focal ODD in the inferior-nasal sector (**Figure 2B**), and a 72-year-old woman with advanced geographic atrophy and moderately severe ODD (**Figure 2C**).

### Patients with ODD can be at a higher risk of developing AMD

To assess whether ODD is associated with increased risk of AMD, we performed a multivariable logistic regression analysis adjusting for age and family history of AMD. Among 94 patients with ODD, 9 (9.6%) had AMD, compared to 3 out of 100 patients without ODD (3.0%) (**Table 1**). In the adjusted model, ODD was associated with higher odds of AMD (AOR = 3.93, 95% CI: 0.89–21.85, *p* = 0.084), although this association did not reach statistical significance. Age and family history were both significant predictors of AMD (age: AOR = 1.12 per year, 95% CI: 1.06–1.22, *p* = 0.001; family history: AOR = 11.95, 95% CI: 2.44–68.64, *p* = 0.003) (**Supplementary Table 1**). The ODD group was significantly younger than the non-ODD group (median age 44 vs. 60 years, *p* = 0.003) (**Table 1**), and AMD cases were primarily observed in older patients (median age: 78, IQR: 73–81). Therefore, the small sample size and the younger age in the ODD group likely limited statistical power and contributed to the wide confidence interval and lack of statistical significance.

**Table 1:** Baseline characteristics of the study cohorts.

	ODD *N* = 94	Non-ODD *N* = 100	*p*-value^1^
AMD	9 (9.6%)	3 (3%)	0.057
Median age (Q1, Q3)	**44** (20, 69)	**60** (44, 69)	0.003
Sex			0.027
Female	60 (64%)	48 (48%)	
Male	34 (36%)	52 (52%)	
Ethnicity			0.14
White	49 (73%)	68 (72%)	
Asian	5 (7.5%)	12 (13%)	
Others	13 (19.5%)	14 (15%)	
Unknown	27	5	
Family History of AMD	11 (14%)	7 (7.0%)	0.13
Unknown	14	0	

^1^Pearson’s chi-squared test; Wilcoxon rank sum test; Fisher’s exact test.

### Patients with ODD-AMD are Caucasians and the majority have a family history of AMD

Analysis of the 10 patients with ODD-AMD showed that all 10 patients were Caucasians and older than 55 years old (**Table 2**). Detailed clinical information about each participant is available online (**Supplementary Table 2**). All patients had bilateral features of ODD. The AMD ranged from early dry AMD to late dry and wet AMD. Extramacular drusen were present in one patient. In addition, seven patients (70%) had a family history of AMD, six of whom were from first-degree relatives. The patients had an adult-onset presentation, with no syndromic features. History of smoking was reported in three patients only.

**Table 2:** Demographics and clinical features of the ODD-AMD subjects.

	ODD-AMD (*n* = 10, eyes = 20)
Patient Demographics	
Age (range)	Median: 75 years (56–91 years)
Sex	F: 70%, M: 30%
Ethnicity	White: 100%
Family history	70% with family history of AMD None with family history of ODD
Disease Severity of the Eyes	
ODD severity	Superficial: 2 (10%) Buried: 4 (20%) Both: 14 (70%)
	Mild (≤ 1 quadrant): 6 (30%) Moderate (2–3 quadrants): 7 (35%) Severe (whole disc): 7 (35%)
AMD stage	Early: 8 (40%) Intermediate: 6 (30%) Advanced GA: 4 (20%) Advanced exudative: 2 (10%)
Ophthalmic Measurements	
BCVA	20/40 or better: 13/20 eyes (65%) Worse than 20/40: 7/20 eyes (35%)
HVF MD (range)	Median: −7.8 dB (−25 to 0.11 dB)
Pattern of vision loss	Vision loss due to optic neuropathy: 4 (40%) Vision loss due to maculopathy: 3 (30%) Both: 3 (30%)
Global peripapillary RNFL thickness (range)	Median: 66 µm (46–127 µm)
Global macular GCC thickness (range)	Median: 64 µm (30–88 µm)
Central macular thickness (range)	Median: 248.5 µm (193–300 µm)

F, female; M, male; AMD, age-related macular degeneration; ODD, optic disc drusen; GA, geographic atrophy; BCVA, best corrected visual acuity; HM, hand motion; HVF MD, Humphrey visual field mean deviation; RNFL, retinal nerve fiber layer; GCC, ganglion cell complex.

The severity of vision loss was diverse and occurred as central vision loss or peripheral field constriction. The visual acuities and field defects are described in **Supplementary Table 2**. The visual acuity was worse than 20/40 in 35% of the eyes. The visual field defects (defined as mean deviation worse than −2 dB) were present in 10 eyes (55.6%).

### Imaging features of ectopic calcification at the optic disc and macula in ODD-AMD

We investigated the different ophthalmic imaging modalities to assess ODD-AMD. Typical features of ODD and AMD were identified bilaterally in the 10 patients. The macular drusen were mainly in association with non-exudative AMD. Four patients showed early AMD with small to medium-sized drusen. One of these also showed scattered peripheral extramacular retinal drusen (**Figure 2A**). Three patients had large (>125 μm) drusen (**Figure 2B**), and two developed geographic atrophy (**Figure 2C**). Signs of neovascularization were apparent in one patient only.

ODD was detectable in eight patients on the en face color fundus photographs (refractile bodies) or FAF imaging (hyper-autofluorescent signal). They were observed in the inferior sector, superonasal sector, and diffusely in different patients (**Figure 2**). ODD was present in at least two quadrants of the optic disc in 70% of the eyes. EDI-OCT was necessary for the detection of ODD in two cases, which showed mild features.

### Ectopic calcification in both optic nerve and retina appears as a hyperreflective outer edge and hyporeflective core

**Figure 3:**
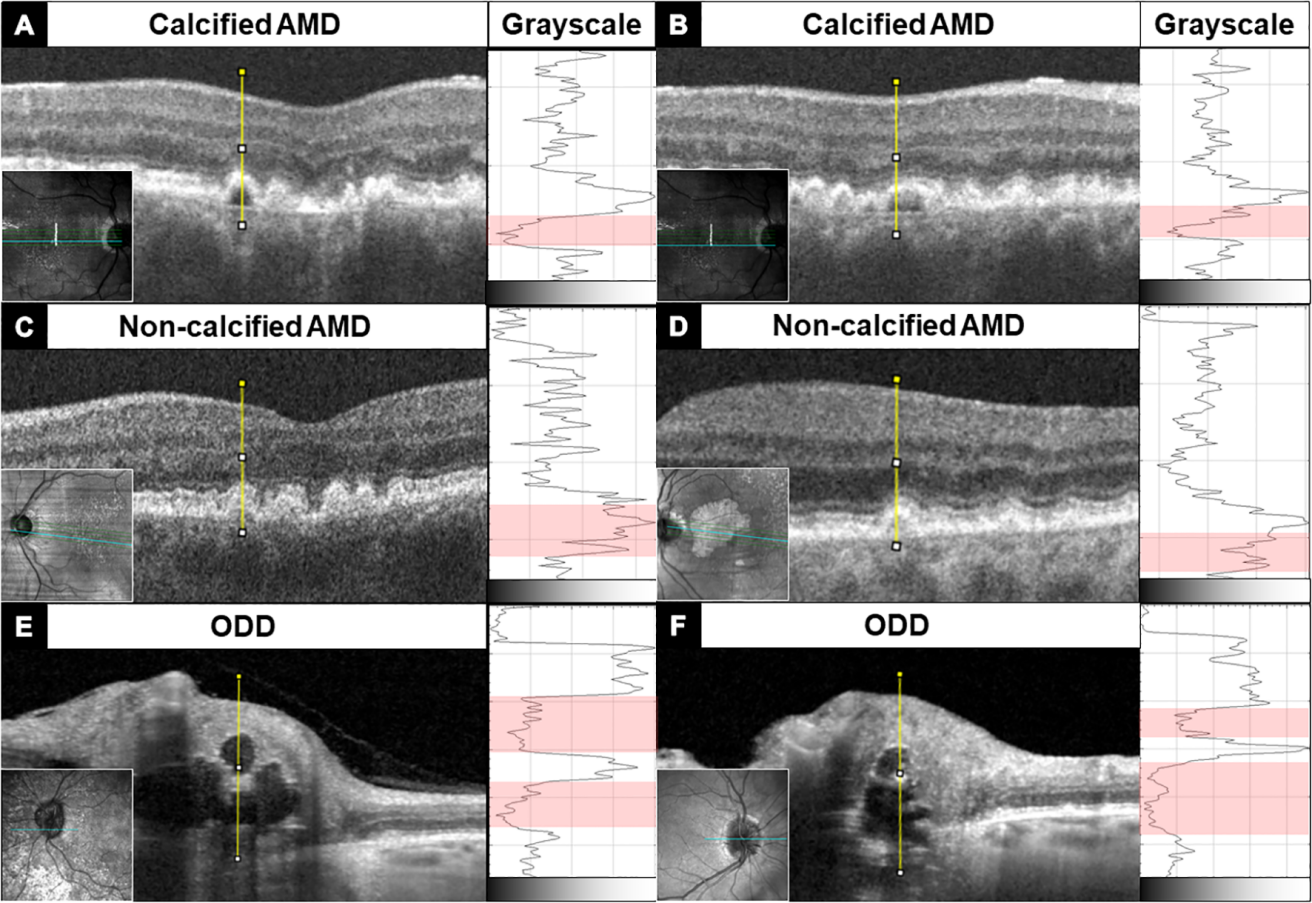
OCT B-scan of macular drusen **(A–D)** and optic disc drusen **(E, F)** showing an abrupt transition from the hyperreflective drusen border to the hyporeflective core —an imaging feature of ectopic calcification. This change in reflectivity profile is quantified along the yellow line through the center of the drusen using ImageJ and displayed to the right of each B-scan in grayscale. The red band in grayscale demarcates the drusen core, which has the lowest reflectivity. Macular drusen have only one small hyporeflective core, while optic disc drusen may have a large hyporeflective core, and multiple calcified deposits are seen stacked on top of each other, rising above the Bruch’s membrane opening.

Typical calcified macular drusen were noted in 40% of patients. **Figure 2** shows an example of ectopic calcification of the optic disc and the macula. The elevated RPE with a hyporeflective core and a hyperreflective cap (**Figure 1A**) represents calcified macular drusen in patients with AMD. A similar pattern in ODD is shown with hyperreflective horizontal lines and signal-poor core (**Figure 1B**). A grayscale plot of the different patterns of drusen appearance on OCT imaging is demonstrated in **Figure 3**. An increased grayscale signal followed by a decreased signal is observed in the calcified AMD and ODD images (**Figures 3A, B, E, F**). This pattern was not apparent in non-calcified AMD drusen (**Figures 3C, D**).

### Calcification in ODD is greater and encompasses the entire drusen deposit while macular drusen exhibit more heterogeneity within the drusen

**Figure 4:**
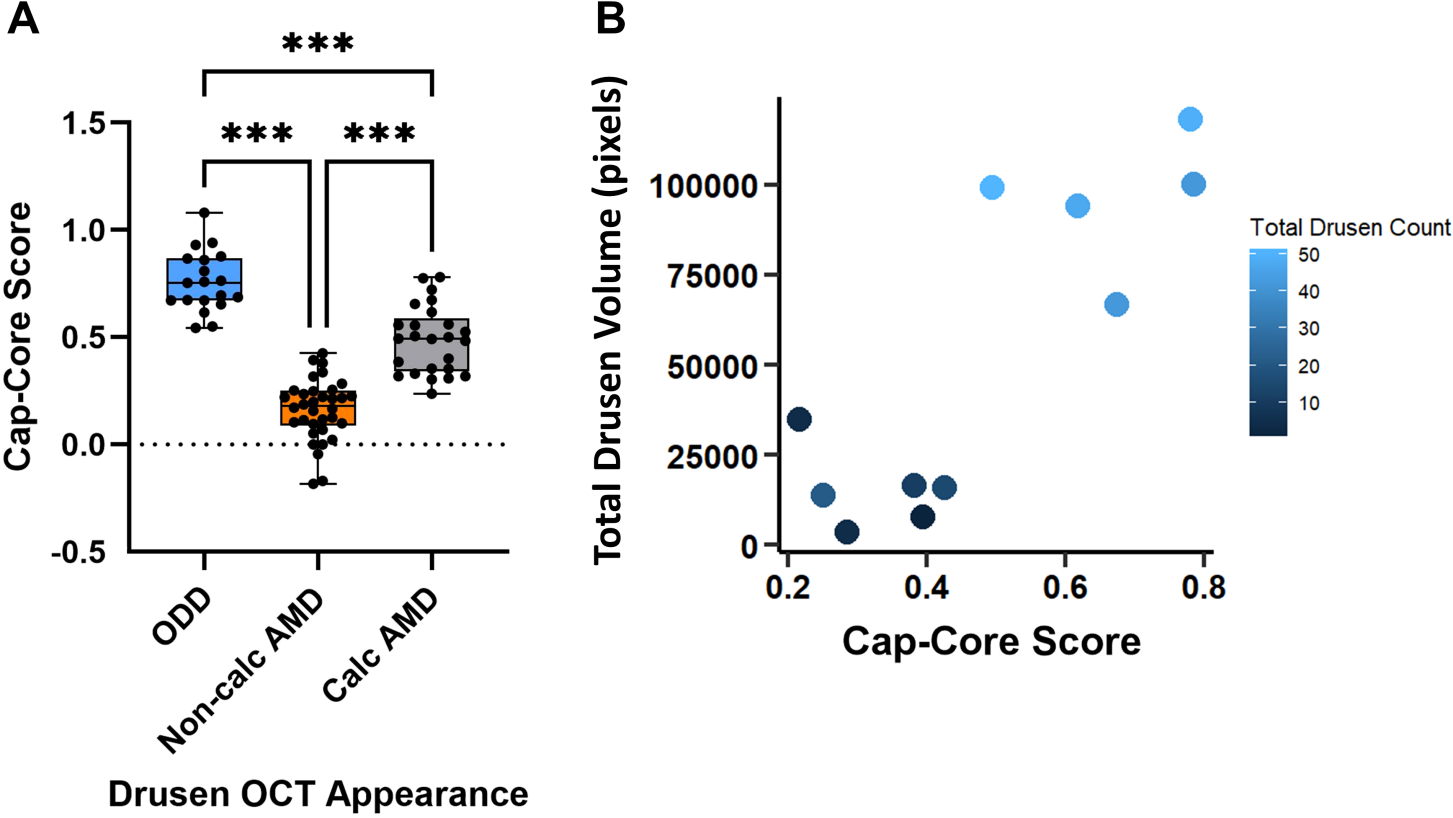
Quantification of the Cap-Core score, in optic disc drusen (ODD), macular drusen without calcification (non-calcified AMD), and macular drusen with calcification (calcified AMD) from 11 ODD-AMD eyes. The Cap-Core score is a measurement of the severity of calcification and defined as the difference in reflectivity between the hyperreflective drusen border and mean drusen core (see Methods for equation). **(A)** Box- and-whisker plot of the Cap-Core score. The central line signifies the mean, and the box delineates the 25th to 75th percentiles (IQR); the whiskers extend to the minimal and maximal values. **(B)** Scatter plot of the macular Cap-Core score and the total macular drusen volume and number of discrete macular drusen deposits (darker means fewer macular drusen). Note that each Cap-Core score is normalized to the vitreous to account for slight variability of signal strength and each symbol is a drusen score measured in a line scan through the drusen center. ***p-value< 0.001 (one-way ANOVA and *post-hoc* Tukey test). Non-calc, noncalcified; Calc, calcified.

When quantifying the difference in reflectivity between the hyperreflective cap and the hyporeflective core, ODD showed the highest mean difference while non-calcified macular drusen had the least difference (**Figure 4A**). The measured means were 0.76 (SD = 0.14) in ODD, 0.5 (SD = 0.16) in the likely calcified macular drusen, and 0.16 (SD = 0.14) in the non-calcified macular drusen. A statistical significance was observed between all three groups (*P*< 0.001). To correlate the macular Cap-Core score with disease burden (based on the number of discrete macular drusen and total area of macular drusen), the drusen with the highest Cap-Core scores were plotted against the total drusen volume and drusen count in each eye (**Figure 4B**). This revealed that macular drusen with a higher Cap-Core score tend to be in eyes with higher drusen count and total drusen volume. Finally, the drusen size was not correlated with its corresponding Cap-Core score in ODD and macular drusen (**Supplementary Figure 5**).

## Discussion

ODD and AMD are among the common eye diseases that impact visual function. The co-existence of the two entities questions the possible relationship and similarities in their pathophysiology. There have been only two cases previously published of a 52-year-old man and a 46-year-old woman with bilateral ODD and macular drusen (8, 9). Here, we found a possibly increased AMD risk among an ODD cohort and highlighted 10 cases of bilateral ODD and concomitant AMD. As patients with ODD present at an earlier age, the possible ODD-AMD association would allow clinicians to identify and monitor these patients for AMD before vision loss ensues.

The odds of AMD were approximately four times higher in the ODD cohort compared to the patients with no ODD. Although this was not statistically significant, the logistic regression model suggested that the age disparity and limited statistical power hindered the ability to detect a significant association, indicating that a larger age-matched cohort can demonstrate a significant association. Both ODD and AMD are most frequently found among Caucasian female patients, which was consistent with our patients (1, 16). In addition, when comparing the patients with ODD-AMD to the typical AMD population, they tend to have the same demographics (age above 70 years and Caucasian population) (17). Smoking is a major risk factor in AMD; however, only three patients in this study reported a history of smoking. More interestingly, the majority of the patients had at least one first-degree family member with an AMD diagnosis. A previous study shows that approximately 20% of patients with AMD report a family history of the disease (18). This suggests that genetic factors may play a more important role in ODD-AMD than environmental factors. Yet, a larger study exploring the role of genetic factors between ODD-AMD and AMD-only is needed. More than 45 genetic loci potentially contributing to AMD development have been identified to date (19). Only recently, Steensberg et al. identified eight genes that might play a role in ODD development (20). The similar patient demography and the genetic component further underscore the need to investigate whether patients with ODD should be screened for AMD at some point. **Table 3** summarizes the current knowledge on ODD and AMD (2, 5, 19, 21–29).

**Table 3:** Several similarities between calcified drusen in ODD and AMD as causes of vision loss.

	ODD	AMD
Reflectance	Refractile extracellular deposits	Refractile extracellular deposits
Drusen Size	50 to 750 µm diameter	<50 to 480 µm diameter
Disease Prevalence	0.4% –3.7%	7.4% –12.3%
Predilection	Caucasians, F>M	Caucasians, F>M
Genetic Factors	Likely polygenic, rarely autosomal dominant	Polygenic, rarely autosomal recessive
Ectopic Calcification	Calcified intra- and extracellular mitochondria in optic nerve	Calcified extracellular deposits between RPE and BM
Drusen Composition	Extracellular calcified deposits in the anterior optic nerve axons	Extracellular deposition of lipofuscin between the RPE and BM
Mechanism of Vision Loss	Vascular > neurodegenerative	Neurodegeneration > vascular
Vascular Complications	NAION, CNV, CRVO, CRAO	Impaired CC flow, CNV

ODD, optic disc drusen; AMD, age-related macular degeneration; F, female; M, male; RPE, retinal pigment epithelium; BM, Bruch’s membrane; NAION, non-arteritic ischemic optic neuropathy; CNV, choroidal neovascularization; CRVO, central retinal vein occlusion; CRAO, central retinal artery occlusion; CC, choriocapillaris.

Pathologically, both ODD and macular drusen involve calcified extracellular deposits, and 4 of our 10 patients with ODD-AMD showed imaging features of the calcification in the macular drusen. Drusen in AMD appear as discrete areas of RPE elevation with variable reflectivity. Calcified drusen have a hyporeflective core with a hyperreflective cap on the image (30). The grayscale plots in our cases quantitatively demonstrated this heterogeneity in the AMD drusen. Overall, the AMD drusen clustered into two distinguished groups despite some outliers, consistent with their appearance on SD-OCT images. Although the pattern of reflectivity was similar between calcified macular drusen and ODD, it remained quantitatively different. This could be attributed to the heterogeneity within the core of the macular drusen, even when they appear to be calcified with the bare eye. Clinical–pathological studies suggest that calcified macular drusen represent a stage of drusen regression and RPE atrophy (28, 31). This is also demonstrated in our study with higher Cap-Core scores showing higher total drusen volume in the eyes.

Several studies assessed the prevalence and features of calcified drusen in AMD using different imaging modalities, reporting a prevalence as high as 42.7% of eyes with AMD (32–34). However, none of these studies reported the number of ODD among their patient population. Hence, a comprehensive evaluation with multimodal imaging should be considered in these patients to detect the prevalence of co-occurrence. Over 100 OCT biomarkers are being studied as AMD prognosis predictors, with hyporeflective drusen cores being among the most clinically relevant ones (35). Given that calcification in macular drusen is a progressive process with variable appearance on OCT images, the Cap-Core score can be a useful measurement for macular drusen calcification, in particular, since it resembles another known form of ectopic calcification, ODD. In addition, there was a trend of higher total drusen volume with higher Cap-Core scores, making it a potential predictor of disease burden if confirmed by a larger study. Still, the grader variability can limit its application. Applying this technique with an automated system can improve its reliability. Lu et al. et al. developed a strategy to automatically segment and quantify calcified drusen using slab-defined en face sub-RPE OCT images, previously described by Liu et al. (32, 36).

Although our findings suggest a possible association between ODD and AMD, several factors limit the conclusion of this study. A primary concern is the wide confidence interval observed in the risk of AMD in patients with ODD, which is expected given the small sample size in this study. However, it remains the largest available database of ODD and represents an important communication to emphasize this association. Although ODD is frequently asymptomatic and underdiagnosed, such an association would be important for the early identification of AMD. A future approach using data from aggregated electronic medical records (i.e., IRIS registry) or using an AMD cohort might allow a larger-scale age-matched study to confirm our findings. Moreover, a detailed comparison between patients with ODD-AMD and those with AMD alone to identify visual outcomes and whether these patients would present earlier was not possible due to the nature of the study. The comparison cohort may also be a limiting factor in this study, as it only included patients from the neuro-ophthalmology clinic, with a high number of ischemic optic neuropathy patients. Although this ensured that they received a formal ophthalmic evaluation, the generalizability of the findings can be limited. Finally, the image analysis was performed by a single grader, which might result in some variability in the drusen analysis.

## Data Availability

The original contributions presented in the study are included in the article/**Supplementary Material**. Further inquiries can be directed to the corresponding author.
