# Supplementary material for: Age-related macular degeneration associated with optic disc drusen
Source: Front Ophthalmol (Lausanne). 2025 Jul 3;5:1620616. doi: 10.3389/fopht.2025.1620616 (PMC12267001; doi:10.3389/fopht.2025.1620616)
Supplement: Supplementary file 1 [file Image1.pdf]

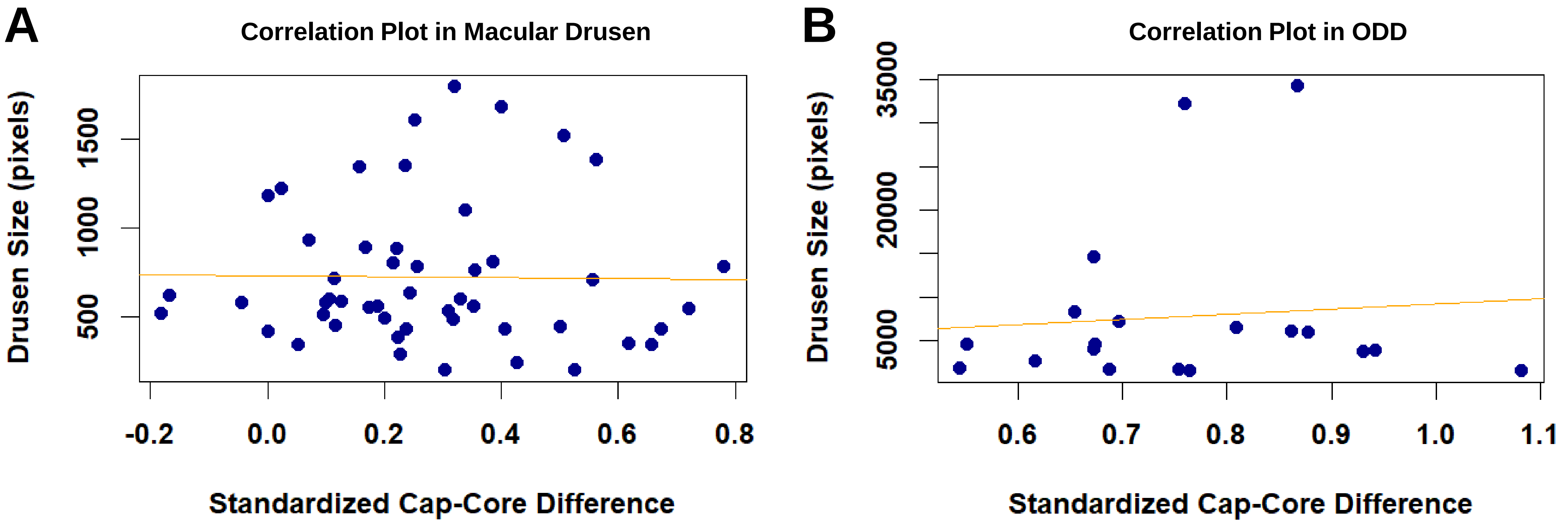

Figure 5 supplemental: (A) Correlation plot of the standardized cap-core difference in macular drusen and its corresponding drusen size. **(B)** Correlation plot of the standardized cap-core difference in ODD and its corresponding drusen size. ODD: Optic Disc Drusen.
