# Supplementary material for: Age-related macular degeneration associated with optic disc drusen
Source: Front Ophthalmol (Lausanne). 2025 Jul 3;5:1620616. doi: 10.3389/fopht.2025.1620616 (PMC12267001; doi:10.3389/fopht.2025.1620616)
Supplement: Supplementary file 2 [file Table1.pdf]

Variable	Coefficient (β)	SE	Wald χ^2	P-value	Adjusted Odds Ratio (AOR)	95% CI
ODD	1.370	0.792	2.99	0.084	3.93	(0.83–18.59)
Age (year)	0.117	0.036	10.71	0.001	1.12	(1.05–1.21)
Family History	2.481	0.833	8.87	0.003	11.95	(2.34–61.15)

Table 1 supplemental. Multivariable Logistic Regression Assessing the Association Between Optic Disc Drusen (ODD) and Age-Related Macular Degeneration (AMD). Adjusted odds ratios (AOR) with 95% confidence intervals are shown for ODD, age, and family history of AMD. Although ODD was associated with higher odds of AMD, the association did not reach statistical significance.
