# Supplementary material for: Age-related macular degeneration associated with optic disc drusen
Source: Front Ophthalmol (Lausanne). 2025 Jul 3;5:1620616. doi: 10.3389/fopht.2025.1620616 (PMC12267001; doi:10.3389/fopht.2025.1620616)
Supplement: Supplementary file 3 [file Table2.pdf]

	Patient 1	Patient 2	Patient 3	Patient 4	Patient 5	Patient 6	Patient 7	Patient 8	Patient 9	Patient 10
**Demographics**										
**Age**	72	77	72	80	73	81	56	66	91	78
**Sex**	Male	Female	Female	Male	Female	Female	Female	Male	Female	Female
**Ethnicity**	White	White	White	White	White	White	White	White	White	White
**Medical History**										
**ODD Presentation**	Peripheral vision loss	Incidental	Peripheral vision loss	Incidental	Peripheral vision loss	Peripheral vision loss	Incidental	Peripheral vision loss	Incidental	Incidental
**AMD**	Early AMD	Intermediate AMD	Advanced GA	Advanced GA	Early AMD	Intermediate AMD	Early AMD	Early AMD	Exudative AMD	Intermediate AMD
**Family History**	AMD (Father)	AD-AMD (Mother and 2 aunts)	None	AMD (Father)	AMD (Mother) Kidney stones and high uric acid	Glaucoma (Father)	AMD (Maternal great aunt)	Optic neuropathy and AMD (Mother and sister)	AMD (Mother and father)	None
**Other Eye Problems**	Cataract Extramacular drusen	Cataract, glaucoma	Cataract	Cataract	Cataract	Cataract	LASIK	AION	Glaucoma	Cataract, AION
**Others**	Malignant fibrohistiocytoma	Lyme disease	Hyperlipidemia, thyroiditis, diverticulosis	Prostate cancer	Kidney stone, hyperuricemia, asthma, migraine	None	Hyperlipidemia, migraine	Kidney stone	Hyperlipidemia, myelodysplasia, cardiovascular diseases	HTN, congestive heart failure, diabetes, dyslipidemia
**Ophthalmic (OD, OS)**										
**BCVA**	20/30, 20/25	20/60, 20/20	20/400, HM	20/40, 20/50	20/20, 20/25	20/30, 20/25	20/25, 20/20	20/25, 20/20	20/100, NLP	20/25, 20/60
**HVF MD (dB)**	-25, -24	-0.60, -0.94	-9.04, -5.10	-	-0.17, - 10.69	0.11, -6.8	-4, -4	-8.4, -7.2	-	-14.48, -15.19
**HVF Grayscale**	Bilateral severe peripheral field constriction	Very subtle changes	OD: Inferonasal arcuate defect OS: Subtle changes	-	OD: Subtle nasal step OS: Moderate constriction	OD: No changes OS: Inferior constriction	Bilateral inferior patchy field loss	OD: Superonasal field loss OS: Inferior altitudinal and nasal field loss	-	OD: Peripheral decrease except temporally OS: Inferior decrease, superior constriction
**Central Macular Thickness**	251, 247	227, 250	234, 280	-	255, 245	289, -	274, 275	294, 300	193, -	216, 203
**RNFL Thickness**	53, 46	66, 85	83, 104	-	86, 57	77, 55	127, 101	71, 66	84, -	51, 51
**GCC Thickness**	52, 31	65, 64	40, 46	-	88, 59	68, 67	66, 67	80, 72	30, -	46, 39

Table 2 supplemental: Clinical Information of Each Patient with Optic Disc Drusen and Age-related Macular Degeneration. AD-AMD: Autosomal dominant age-related macular degeneration, GA: Geographic atrophy, LASIK: Laser-assisted in situ keratomileusis, AION: Anterior ischemic optic neuropathy, OD: Right eye, OS: Left eye, BCVA: Best corrected visual acuity, HM: Hand motions, HFV: Humphrey visual field, MD: Mean deviation, RNFL: Retinal nerve fiber layer, GCC, Ganglion cell complex. “-”: Missing information.
